# Supplementary material for: Etiology, characteristics and occurrence of heart diseases in rural Lesotho (ECHO-Lesotho): A retrospective echocardiography cohort study
Source: PLoS One. 2022 Dec 15;17(12):e0278406. doi: 10.1371/journal.pone.0278406 (PMC9754242; doi:10.1371/journal.pone.0278406)
Supplement: S1 Text — (DOCX) [file pone.0278406.s001.docx]

**SUPPLEMENT**

**Etiology, characteristics and occurrence of heart diseases in rural Lesotho (ECHO-Lesotho): A retrospective echocardiography cohort study**

**S1 Text:** Echocardiography details

Echocardiography was performed on a Philips ClearVue 350 ultrasound machine (Philips. Koninklijke. USA), using an S4-1 active phased array transducer, performing two-dimensional, time motion, continuous wave, pulsed wave, color doppler and tissue doppler imaging (TDI). The assessments of ventricular and atrial dimensions, right and left systolic and diastolic function, valvular function, regional wall abnormalities and pericardial aspect were conducted according to international recommendations [1–6]. The end-systolic and end-diastolic left ventricular (LV) volumes and the LV ejection fraction (LVEF) were calculated using the Simpson method of discs [1]. The LV diastolic function and the pressure of the left atrium (LAP) were estimated with a multiparametric method using mitral inflow assessment (E-wave/A-wave), E-wave velocity, left atrium (LA) volume index, tricuspid regurgitation peak of velocity, mitral septal and medial annulus (e’), and E-wave/e’ ratio [2]. In case of severe valvular mitral diseases, atrial fibrillation, and hypertrophic cardiomyopathy, isovolumic relaxation time and difference between A-wave pulmonal and A-wave mitral were considered [2]. According to this assessment, LV end diastolic filling pressure was determined as low, high, or indeterminable. Right ventricle (RV) systolic function was estimated by measuring the Tricuspid Annular Plan Systolic Excursion (TAPSE), the RV systolic excursion velocity by TDI and the fractional area change (FAC) [3]. For the right atrium (RA) size we considered the RA area, but for LA, we used volume index. The RA pressure was estimated using the inferior vena cava diameter and the presence of its inspiratory collapse [3]. Pulmonary artery systolic pressure was estimated by tricuspid regurgitation velocity whenever it was possible. If tricuspid regurgitation was not present, then it was measured by the mean pulmonary artery considering RV outflow doppler acceleration time, and pulmonary artery diastolic pressure of the pulmonic regurgitation [3]. Severity of aortic stenosis was measured and interpreted using the valve area, transvalvular gradient of pressure, aortic transvalvular peak of velocity and the permeability index [4]. For the assessment of the mitral valvular stenosis, planimetry, pressure half time, and mean gradient were used [4]. The severity of valve regurgitation was quantified by measuring indirect and direct signs, using the proximal isovelocity surface area method [5].

**References**

1. Lang RM, Badano LP, Mor-Avi V, Afilalo J, Armstrong A, Ernande L, et al. Recommendations for cardiac chamber quantification by echocardiography in adults: an update from the American Society of Echocardiography and the European Association of Cardiovascular Imaging. J Am Soc Echocardiogr. 2015 Jan;28(1):1-39.e14.

2. Nagueh SF, Smiseth OA, Appleton CP, Byrd BF, Dokainish H, Edvardsen T, et al. Recommendations for the Evaluation of Left Ventricular Diastolic Function by Echocardiography: An Update from the American Society of Echocardiography and the European Association of Cardiovascular Imaging. J Am Soc Echocardiogr. 2016 Apr;29(4):277–314.

3. Rudski LG, Lai WW, Afilalo J, Hua L, Handschumacher MD, Chandrasekaran K, et al. Guidelines for the echocardiographic assessment of the right heart in adults: a report from the American Society of Echocardiography endorsed by the European Association of Echocardiography, a registered branch of the European Society of Cardiology, and the Canadian Society of Echocardiography. J Am Soc Echocardiogr. 2010 Jul;23(7):685–713; quiz 786–8.

4. Baumgartner H, Hung J, Bermejo J, Chambers JB, Evangelista A, Griffin BP, et al. Echocardiographic assessment of valve stenosis: EAE/ASE recommendations for clinical practice. J Am Soc Echocardiogr. 2009 Jan;22(1):1–23; quiz 101–2.

5. Zoghbi WA, Enriquez-Sarano M, Foster E, Grayburn PA, Kraft CD, Levine RA, et al. Recommendations for evaluation of the severity of native valvular regurgitation with two-dimensional and Doppler echocardiography. J Am Soc Echocardiogr. 2003 Jul;16(7):777–802.

6. Pettersen MD, Du W, Skeens ME, Humes RA. Regression equations for calculation of z scores of cardiac structures in a large cohort of healthy infants, children, and adolescents: an echocardiographic study. J Am Soc Echocardiogr. 2008 Aug;21(8):922–34.
