# Supplementary material for: Etiology, characteristics and occurrence of heart diseases in rural Lesotho (ECHO-Lesotho): A retrospective echocardiography cohort study
Source: PLoS One. 2022 Dec 15;17(12):e0278406. doi: 10.1371/journal.pone.0278406 (PMC9754242; doi:10.1371/journal.pone.0278406)
Supplement: S1 Table — (DOCX) [file pone.0278406.s002.docx]

**SUPPLEMENT**

**Etiology, characteristics and occurrence of heart diseases in rural Lesotho (ECHO-Lesotho): A retrospective echocardiography cohort study**

**S1 Table:** Echocardiography findings of patients hospitalized due to heart failure

|  | N=126 |
| --- | --- |
| **LV size** |  |
| - Normal size | 60 (47.6%) |
| - LV dilatation | 30 (23.8%) |
| - LV hypertrophy | 36 (28.6%) |
| **LV systolic function** |  |
| - Normal systolic LV function (LVEF ≥ 54%) | 75 (59.5%) |
| - Mild systolic heart failure (LVEF 41-53%) | 11 (8.7%) |
| - Moderate systolic heart failure (LVEF 30-40%) | 12 (9.5%) |
| - Severe systolic heart failure (LVEF < 30%) | 28 (22.2%) |
| **Diastolic relaxation impairment of LV** |  |
| - None | 41 (32.5%) |
| - Grade 2 or 3 | 39 (31.0%) |
| - Diastolic function not determinable | 46 (36.5%) |
| **RV size** |  |
| - Normal size | 23 (18.3%) |
| - RV dilatation | 65 (51.6%) |
| - RV dilatation and hypertrophy | 38 (30.2%) |
| **RV systolic function** |  |
| - Normal | 70 (55.6%) |
| - Impaired | 56 (44.4%) |
| **Pulmonary hypertension** |  |
| - No - Yes, due to the left sided heart disease | 4 (3.2%)  84 (66.7%) |
| - Yes, due to other cause (pulmonary) | 38 (30.2%) |
| **Valvular disease** |  |
| - No - Yes, aortic regurgitation, moderate or severe | 78 (61.9%)  6 (4.8%) |
| - Yes, aortic stenosis, moderate or severe | 2 (1.6%) |
| - Yes, mitral regurgitation, moderate or severe | 16 (12.7%) |
| - Yes, mitral stenosis, moderate or severe | 4 (3.2%) |
| - Yes, tricuspid regurgitation, moderate or severe | 19 (15.1%) |
| - Yes, pulmonal stenosis, moderate or severe | 1 (0.8%) |
| **Pericardium** |  |
| - Significant pericardial effusion (>10mm) | 39 (31.0%) |
| - Pericarditis - Normal | 6 (4.8%)  81 (64.3%) |

Abbreviations: LV (left ventricle), LVEF (left ventricular ejection fraction), RV (right ventricle)
