# Supplementary material for: Etiology, characteristics and occurrence of heart diseases in rural Lesotho (ECHO-Lesotho): A retrospective echocardiography cohort study
Source: PLoS One. 2022 Dec 15;17(12):e0278406. doi: 10.1371/journal.pone.0278406 (PMC9754242; doi:10.1371/journal.pone.0278406)
Supplement: S2 Table — (DOCX) [file pone.0278406.s003.docx]

**SUPPLEMENT**

**Etiology, characteristics and occurrence of heart diseases in rural Lesotho (ECHO-Lesotho): A retrospective echocardiography cohort study**

**S2 Table.** Association between characteristics and most common etiology for heart failure

|  |  | **Univariate logistic regression** | | | **Multivariate logistic regression**  **(LR chi2=61.89, *P*<0.001)** | | |
| --- | --- | --- | --- | --- | --- | --- | --- |
| **Characteristic** | **N (%)** | **Odds Ratio (95% CI)** | **β coefficient** | ***P* value** | **Adjusted Odds Ratio (95% CI)** | **β coefficient** | ***P* value** |
| **Female** (vs. male) | 70 (56) | 0.08 (0.03-0.24) | -2.49 | <0.001 | .34 (.08-1.45) | -1.07 | 0.146 |
| **Age** (per year), median (IQR) | 66 (54-76) | 1.02 (0.99-1.05) | 0.02 | 0.146 |  |  |  |
| **Hypertensive** (vs. non-hypertensive) | 77 (61) | 0.26 (0.12-0.62) | -1.32 | 0.002 | 2.72 (.55-13.48) | 1.00 | 0.219 |
| **Diabetic** (vs. non-diabetic) | 25 (20) | 0.34 (0.09-1.22) | -1.08 | 0.097 |  |  |  |
| **HIV** (vs. non-HIV) | 17 (13) | 3.15 (1.10-9.04) | 1.17 | 0.033 |  |  |  |
| **History of TB** (vs. no history of TB) | 19 (15) | 13.84 (4.43-43.28) | 2.63 | <0.001 | 6.25 (1.24- 31.48) | 1.83 | 0.026 |
| **Taking cardiac medication** (vs. not taking any cardiac medication) | 82 (65) | 0.42 (0.19-0.97) | -0.86 | 0.041 |  |  |  |
| **Smoking** (vs. not smoking) | 18 (14) | 2.11 (0.74-6.02) | 0.75 | 0.162 |  |  |  |
| **Former mine worker** (vs. not former mine worker) | 29 (23) | 37.48 (12.10-116.07) | 3.62 | <0.001 | 24.65 (5.06-120.10) | 3.20 | <0.001 |
